# Stone-Dominant Renal Phenotype Without Nephrocalcinosis in *FAM20A*-Related Enamel Renal Syndrome

**DOI:** 10.1016/j.ekir.2026.103783

**Published:** 2026-01-19

**Authors:** Neriman Sıla Koç

**Affiliations:** 1 Division of Nephrology, Department of Internal Medicine, Ankara Etlik City Hospital, Ankara, Türkiye

To the Editor:

I read with great interest the article by Cogal *et al.*,^1^ in which the authors convincingly demonstrate that monogenic urinary stone disease is characterized by substantial genetic and phenotypic heterogeneity and that reliance on clinical or biochemical features alone may result in missed or delayed diagnoses. Their findings strongly support the use of broad targeted genetic screening in patients suspected of monogenic urinary stone disease or nephrocalcinosis.

I would like to extend this concept of phenotypic heterogeneity by describing a stone-dominant renal phenotype without nephrocalcinosis in patients with *FAM20A*-related enamel renal syndrome**,** a disorder classically associated with diffuse medullary nephrocalcinosis.

Enamel renal syndrome, caused by biallelic *FAM20A* mutations, is classically characterized by amelogenesis imperfecta and renal calcifications.^2^ From a nephrological standpoint, nephrocalcinosis has been considered the hallmark renal manifestation and has been reported in nearly all affected individuals in the largest cohorts.^3^ Nephrolithiasis, when described, usually coexists with medullary nephrocalcinosis rather than representing a dominant feature.^2^^,^^4^

In contrast, I evaluated 2 adult brothers with genetically confirmed enamel renal syndrome who presented with recurrent calcium-based nephrolithiasis in the absence of radiological nephrocalcinosis. Both patients had characteristic dental findings consistent with amelogenesis imperfecta (Figure 1). Genetic testing identified a homozygous pathogenic copy number deletion involving exon 4 of *FAM20A* ***(****NM_017565)*. Noncontrast abdominal computed tomography demonstrated multiple discrete calculi localized to the renal calyces, without diffuse or symmetric medullary calcification (Figure 1).

**Figure 1:**
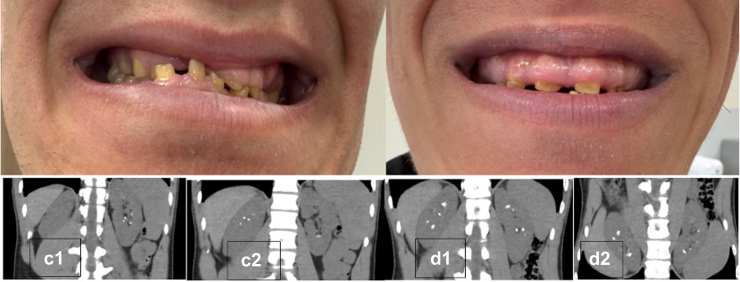
(a and b) Dental photographs demonstrating generalized enamel hypoplasia consistent with amelogenesis imperfecta in both siblings. (c_1-_c_2,_ d_1_, and d_2_) Noncontrast abdominal computed tomography images showing bilateral renal calculi in both cases, with preserved renal parenchyma and absence of cortical or medullary nephrocalcinosis.

Metabolic evaluation showed hypocalciuria and marked hypocitraturia in both patients, with normal urinary oxalate and phosphate excretion. Serum calcium and phosphate levels were within reference ranges, and distal renal tubular acidosis was excluded (Supplementary Table S1). This biochemical profile suggests that impaired urinary crystal inhibition, rather than increased calcium load, may represent a key mechanism underlying stone formation in *FAM20A*-related disease.

Consistent with the observations of Cogal *et al.*,^1^ these findings illustrate that monogenic urinary stone disease represents a continuum of renal phenotypes. I propose that stone-dominant enamel renal syndrome without nephrocalcinosis constitutes an expanded renal phenotype, emphasizing that the absence of classical imaging features should not preclude consideration of monogenic disease. Moreover, my observations suggest that nephrolithiasis without nephrocalcinosis may represent an alternative or early renal manifestation of *FAM20A*-related disease, underscoring the importance of systematic renal imaging and metabolic evaluation in patients with enamel renal disorders, even in the absence of overt renal symptoms.

## Supplementary Material

Supplementary File (PDF) **Table S1.** Comparative metabolic profiles showing hypocalciuria and hypocitraturia with preserved renal function.

## Footnotes

Supplementary File (PDF)
**Table S1.** Comparative metabolic profiles showing hypocalciuria and hypocitraturia with preserved renal function.
