# Supplementary material for: Stone-Dominant Renal Phenotype Without Nephrocalcinosis in FAM20A-Related Enamel Renal Syndrome
Source: Kidney Int Rep. 2026 Jan 19;11(4):103783. doi: 10.1016/j.ekir.2026.103783 (PMC12915170; doi:10.1016/j.ekir.2026.103783)
Supplement: Supplementary File (PDF) — Table S1. Comparative metabolic profiles showing hypocalciuria and hypocitraturia with preserved renal function. [file mmc1.pdf]

Table S1. Comparative metabolic profiles showing hypocalciuria and hypocitraturia with preserved renal function.

Parameter	Case 1	Case 2
**Age (years)**	25	22
**Gender**	Male	Male
**Serum creatinine (mg/dL)**	0.89	0.74
**eGFR (mL/min/1.73 m²)**	119	131
**Serum calcium (mg/dL)**	9.5	9.4
**Serum phosphorus (mg/dL)**	2.9	2.8
**Serum sodium (mmol/L)**	140	141
**Serum potassium (mmol/L)**	4.5	4.6
**Serum uric acid (mg/dL)**	5.6	6
**Serum bicarbonate (mmol/L)**	23.2	25
**Urine pH**	5.0	5.0
**24-h urinary calcium (mg/day)**	*55**	*24.1**
**24-h urinary citrate (mg/day)**	*194**	*157**
**24-h urinary oxalate (mg/day)**	30	27
**24-h urinary phosphate (mg/day)**	674	917

** Abnormal values are indicated by italics and an asterisk.*
